# A shared coevolutionary history does not alter the outcome of coalescence in experimental populations of *Pseudomonas fluorescens*

**DOI:** 10.1111/jeb.13394

**Published:** 2018-11-12

**Authors:** Meaghan Castledine, Angus Buckling, Daniel Padfield

**Affiliations:** ^1^ Centre for Ecology and Conservation College of Life and Environmental Sciences University of Exeter Penryn Cornwall UK; ^2^ Environment and Sustainability Institute University of Exeter Penryn Cornwall UK

**Keywords:** coalescence, coevolution, local adaptation, niche‐packing, parallel evolution, *Pseudomonas fluorescens*

## Abstract

Community coalescence, the mixing of multiple communities, is ubiquitous in natural microbial communities. During coalescence, theory suggests the success of a population will be enhanced by the presence of species it has coevolved with (relative to foreign species), because coevolution will result in greater resource specialization to minimize competition. Thus, more coevolved communities should dominate over less coevolved communities during coalescence events. We test these hypotheses using the bacterium *Pseudomonas fluorescens* which diversifies into coexisting niche‐specialist morphotypes. We first evolved replicate populations for ~40 generations and then isolated evolved genotypes. In a series of competition trials, we determined if using coevolved versus random genotypes affected the relative performance of “communities” of single and multiple genotypes. We found no effect of coevolutionary history on either genotype fitness or community performance, which suggests parallel (co)evolution between communities. However, fitness was enhanced by the presence of other genotypes of the same strain type (wild‐type or an isogenic strain with a LacZ marker; the inclusion of the latter necessary to distinguish genotypes during competition), indicative of local adaptation with respect to genetic background. Our results are the first to investigate the effect of (co)evolution on the outcome of coalescence and suggest that when input populations are functionally similar and added at equal mixing ratios, the outcome community may not be asymmetrically dominated by either input population.

## 1 INTRODUCTION

Community coalescence, where two or more communities come into contact, is ubiquitous in the microbial world (Rillig et al., 2015). The mixing of multiple communities occurs regularly due to natural (e.g., leaves falling and the mixing of aquatic and terrestrial communities during flooding events) and human‐induced (e.g., movement of material during horticulture and farming and release of human‐made industrial waste into aquatic water bodies) processes (Rillig et al., 2015). Despite its ubiquity, research into the mechanisms that govern, and the outcome of, community coalescence and multi‐species invasions is only beginning to be addressed (Lu, Sanchez‐Gorostiaga, Tikhonov, & Sanchez, 2018; Rillig et al., 2015; Rivett et al., 2018; Tikhonov, 2016). Communities arising from coalescence can be chimeric (a combination of species from both communities; Livingston, Jiang, Fox, & Leibold, 2013; Rummens, De Meester, & Souffreau, 2018) or asymmetric (dominance of a singular community; Freilich et al., 2011; Gilpin, 1994; Guo, Harstall, Louie, Veldhuyzen Van Zanten, & Dieleman, 2012; Livingston et al., 2013; Ridaura et al., 2014; Rillig et al., 2015; Sierocinski et al., 2017; Vermeij, 1991). Understanding the ecological and evolutionary mechanisms that underpin the outcome of community coalescence is critical for environmental (LaRue, Chambers, & Emery, 2017), medical (He, McLean, Guo, Lux, & Shi, 2014; Lloyd‐Price, Abu‐Ali, & Huttenhower, 2016) and biotechnological (Rillig, Tsang, & Roy, 2016; Sierocinski et al., 2017) research.

Previous work on multi‐species invasions suggests that the outcome of community coalescence is dependent on the balance between competition within and between each input community (Rivett et al., 2018). Niche‐packing, the partitioning of resource use between members within a community, and functional diversity, the range of niches filled within a community, could both contribute to asymmetric outcomes during community coalescence. Niche‐packed, more functionally diverse, communities can be more resistant to invasion through having fewer vacant niches that invaders can occupy (Elton, 1958; Hodgson, Rainey, & Buckling, 2002; Levine & D'Antonio, 1999). Theory suggests that the presence of other community members, within niche‐packed communities, can enhance the success of a species’ population (Tikhonov, 2016): a process termed “ecological co‐selection” (Lu et al., 2018; Sierocinski et al., 2017). For example, when methanogenic communities are coalesced in bioreactors, the subsequent mixture is dominated by the most productive community (Sierocinski et al., 2017). In addition, invasion experiments on natural isolates demonstrate that the presence of other community members aids recruitment of species into a coalesced community (Lu et al., 2018).

A key question remains as to what determines the extent of niche‐packing within communities. A shared coevolutionary history is likely to play a crucial role. When species compete, selection increases resource specialization and reduces niche overlap, which can result in adaptive radiations (Gillespie, 2004; Rainey & Travisano, 1998; Schluter, 2000) and character displacements (Ellis, Traverse, Mayo‐Smith, Buskirk, & Cooper, 2015; Grant & Grant, 2006; Stuart et al., 2014). Individuals or species that occupy vacant niches are selected for to reduce within‐community competition (Schluter, 2000). Consequently, communities that have a history of coexistence may experience less within‐community competition, allowing them to dominate during coalescence events (Livingston et al., 2013; Tikhonov, 2016). However, a direct test of the role of coevolution in shaping the outcome of community coalescence is lacking.

**Figure 1:**
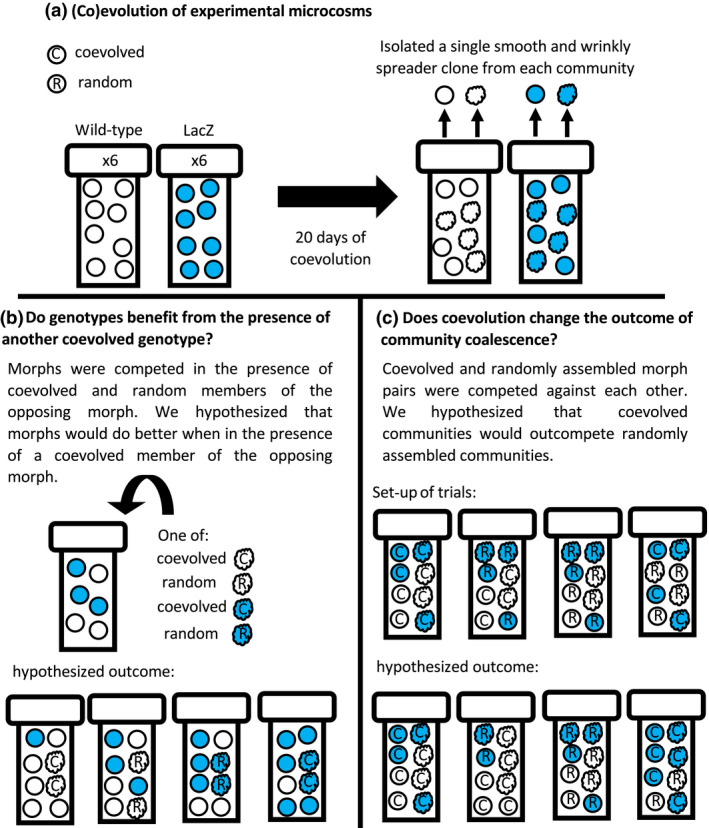
Overview of experimental design and competition trials. (a) Experimental populations of *Pseudomonas fluorescens* were maintained for 20 days. Colonies of WS and SM morphs were picked at the final time point. (b) Isolated morphs were used to see whether the presence of an additional morph with a shared coevolutionary history increased the morphotype relative fitness. (c) Whether a shared coevolutionary history improved community performance during community coalescence was tested by competing wild‐type and LacZ communities where both morphs were present in all combinations of coevolutionary history

Here, we use isogenic populations of the bacterium *Pseudomonas fluorescens* SBW25 to examine the importance of coevolution to the outcome of community coalescence. When grown in static conditions, *P. fluorescens* diversifies into resource‐specialist morphotypes; the two most common being the wrinkly spreader (WS), which occupies the air–liquid interface, and the smooth morph (SM), which occupies the liquid niche (Rainey & Travisano, 1998). These evolved populations have characteristics expected of simple niche‐packed communities, in that the members stably coexist [as a result of negative frequency‐dependent selection (Rainey & Travisano, 1998)] and diversity increases productivity and invasion resistance (Hodgson et al., 2002). Importantly, diversification of WS and SM occurs to reduce competition between their respective niches, which previously resulted in coevolutionary dynamics within 50 generations in similar experimental conditions (Zhang, Buckling, Ellis, & Godfray, 2009). Therefore, this system is useful for testing community coalescence theory, and we refer to our populations as communities for the remainder of the manuscript. To distinguish between communities after coalescence, we used a genetically identical mutant with a LacZ genetic marker which turns colonies blue when grown on agar containing X‐gal (Zhang & Rainey, 2007). Using this model system, we manipulate coevolutionary history to determine its impacts on conspecific fitness and community coalescence (Figure 1). Specifically, in correspondence with the niche‐packing hypothesis, we predict that: (a) the relative fitness of a genotype will increase in the presence of the functionally distinct genotype it coevolved with (Figure 1b); and (b) input communities that contain morphotypes that have coevolved together will be more successful than randomly assembled communities (Figure 1c).

## 2 MATERIALS AND METHODS

### 2.1 Experimental set‐up and isolation of niche specialists

Six replicate populations of *P. fluorescens* SBW25 (wild‐type) and six populations of a genetically marked SBW25 LacZ strain were cultivated in 6 ml of King's medium B (KB) and kept in static conditions at 28°C in 25‐ml glass microcosms with loosened plastic lids. Serial 100‐fold dilutions (60 μl into 6 ml KB) took place every 4 days for a total of 20 days (Figure 1a). After each serial dilution, samples of each culture were cryogenically frozen at −80°C in glycerol (final concentration: 25%). Different niche specialists of each replicate population were isolated by plating cultures after 20 days onto KB agar and incubating for 2 days at 28°C. Single clones of the most common morphotypes, wrinkly spreader and smooth, were picked for each replicate and cryogenically frozen at −80°C in 25% glycerol (Figure 1a). Twenty‐four hours prior to being used in experimental trials, morphotypes were grown in monoculture in KB at 28°C on an orbital shaker at 180 r.p.m to achieve high cell densities.

### 2.2 Experimental design and competition assays

Across the 12 replicate populations (six wild‐type and six LacZ), microcosms were paired between and within strains. This resulted in three blocks of four communities (two wild‐type and two LacZ populations in each block) from which coevolved and randomly assembled communities for the competition trials were designed. Within each block, competition trials were designed for all combinations of coevolutionary history (coevolved or randomly assembled) and levels of morphotype diversity (i.e., 1 vs. 1, 2 vs. 1 and 2 vs. 2; Figure 1b and Table S1), resulting in a total of 132 trials. Random communities were created by swapping reciprocal WS and SM morphotypes within a block (i.e., WS from LacZ community 1 and SM from LacZ community 2). Coevolved communities were reconstructed from WS and SM morphotypes isolated from the same population. This ensured the effect of coevolution could be measured whilst controlling for differences in genotypic diversity that also influences invasibility (Jousset, Schulz, Scheu, & Eisenhauer, 2011).

Communities for competition trials were constructed at 1:1 ratios from overnight monocultures of WS and SM morphotypes. In each trial, competing communities were inoculated at ratios of 1:1 (30 μl of both wild‐type and LacZ communities) with optical density normalized to ~0.1 OD_600_ in the inoculate stock. Inoculate stocks were then cryogenically frozen in glycerol (25% final concentration) and subsequently plated onto KB agar containing 40 μg/ml of X‐gal (5‐bromo‐4‐chloro‐3‐indolyl‐β‐D‐galactopyranoside). Competition trials were inoculated into KB medium under the same conditions as the initial serial dilutions. After 4 days, cultures were frozen as above and subsequently plated on agar containing X‐gal where LacZ colonies can be distinguished by their blue colour. Morphotype density (CFUs/ml) was calculated by counting the number of colony‐forming units (CFUs) after 2 days of growth at 28°C. Relative fitness was defined as the fitness of one LacZ morph versus the same wild‐type morph (i.e., LacZ SM vs. wild‐type SM). Relative community performance was defined as the performance of the whole LacZ community relative to the competing wild‐type community. Both relative fitness and community performance were calculated from the ratio of the estimated Malthusian parameters, *m*_LacZ_:*m*_wild‐type_, which were calculated as *m *= ln(*N*_1_/*N*_0_), where *N*_1_ is the final density and *N*_0_ is the starting density (Lenski, Rose, Simpson, & Tadler, 1991).

### 2.3 Statistical analyses

Competition trials were analysed in *R* (v 3.4.3; R Core Team, 2013). As the experimental design resulted in more replicates of randomly assembled versus single genotype than coevolved versus single genotype pairings (Table S1), the relative fitness of equivalent trials (e.g., LacZ SM1 vs. wild‐type WS1 and SM2, and LacZ SM1 vs. wild‐type WS2 and SM1) were averaged (by taking the mean) to yield balanced, more interpretable, data sets where necessary. Separate linear mixed‐effect models, using the package “*lme4*” (Bates, Maechler, Bolker, & Walker, 2014), were used to test each hypothesis as a single, global model incorporating morphotype diversity and coevolutionary history of both LacZ and wild‐type communities would include impossible combinations of factors (e.g., there is no coevolved wild‐type when there is only one wild‐type morphotype).

First, we tested whether the presence of a coevolved genotype increased the relative fitness of a focal genotype. To do this, we used the competition trials where there was a single morphotype against both morphotypes for either LacZ or wild‐type communities, and when the same morphotype competed in the absence of any other morphotype. We analysed whether relative fitness was influenced by coevolutionary history or the presence of another community member of the same strain per se. Morphotype (either smooth or wrinkly spreader) was added as a potentially interacting factor to look for asymmetric effects between morphs. We then selected trials where SM and WS morphotypes were present in both LacZ and wild‐type communities to investigate whether coevolved communities were more successful than randomly assembled communities during community coalescence. In this model, coevolutionary history of LacZ and wild‐type communities were included as potentially interacting factors.

For all analyses, a random effect was included to account for the hierarchical structure of the data (communities within blocks). Model simplification was done using likelihood ratio tests, and Tukey's post hoc multiple comparison tests were done on the most parsimonious model using the *R* package “*emmeans*” (Lenth, 2018). We also checked whether the strain (LacZ or wild‐type) influenced individual‐level morphotype relative fitness and relative community performance by averaging over the effects of other factors of the final model from the morphotype‐level analysis. An average relative fitness >1 with confidence intervals that do not overlap 1 indicated that the LacZ strain had a significantly greater fitness than the wild‐type strain.

## 3 RESULTS

**Figure 2:**
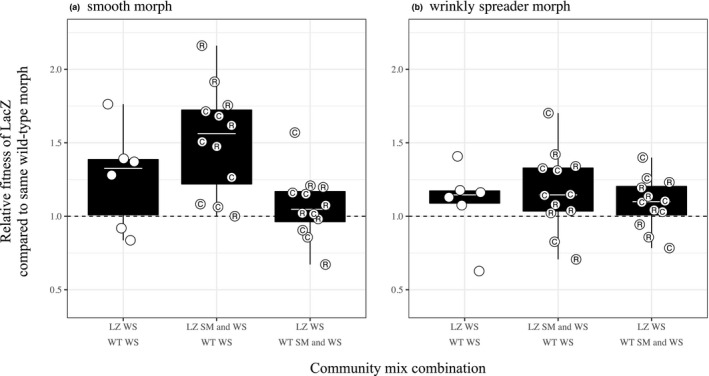
Relative fitness of (a) LacZ smooth (SM) and (b) LacZ wrinkly spreader (WS) morphotypes when competed in the absence (first bar) and in the presence of additional coevolved or random LacZ or wild‐type genotypes. Points represent independent competition trials in the absence or in the presence of an additional coevolved (circle with C) or random (circle with R) genotype. A shared coevolutionary history had no impact on the outcome of community coalescence, but LacZ genotypes were fitter than wild‐type genotypes. Dotted line (*y *=* *1) indicates where relative fitness, between strains, is equal. Tops and bottoms of the bars represent the 75th and 25th percentiles of the data, the white lines are the medians, and the whiskers extend from their respective hinge to the smallest or largest value no further than 1.5* interquartile range

The presence of coevolved genotypes did not alter the relative fitness of genotypes. Specifically, the outcome of competition between two genotypes (with the same morphotype; either SM or WS) from different communities was not influenced by the presence of coevolved genotypes versus a random genotype or the absence of additional genotypes (Figure 2; Tukey's post hoc multiple comparisons: all *p*‐values > 0.05). As the presence of coevolved genotypes had no impact on genotypic relative fitness, we then determined whether LacZ/wild‐type genotypic fitness was influenced by the presence of additional LacZ/wild‐type genotypes per se. The results were inconsistent across genotypes (Figure 2, Table S3), but the relative fitness of LacZ SM (compared to wild‐type SM) was significantly greater in the presence of LacZ WS (relative fitness = 1.52, 95% CI = 1.37–1.67) compared to when wild‐type WS was the additional morphotype (relative fitness = 1.07, 95% CI = 0.91–1.22). However, the addition of an additional WS morphotype (either LacZ or wild‐type WS) did not significantly increase or decrease fitness of LacZ SM compared to when there were no additional morphs present (i.e., LacZ SM vs. wild‐type SM; Figure 2a). Relative fitness of wrinkly spreaders was independent of the strain background of any additional genotypes that were present (Tukey's post hoc multiple comparisons, all *p*‐values > 0.05; Figure 2b). In addition to the benefit received by LacZ SM from the presence of LacZ WS, LacZ genotypes were fitter overall (relative fitness of LacZ SM: 1.28, 95% CI: 1.14–1.43; relative fitness of LacZ WS: 1.12, 95% CI: 0.97–1.27).

**Figure 3:**
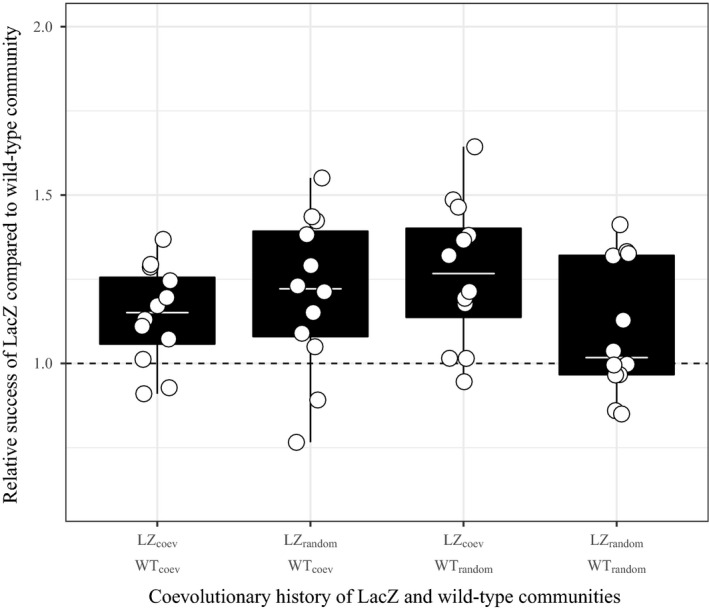
Relative performance of LacZ communities where wrinkly spreader and smooth morph are present for both competing communities. Competition trials included all combinations of coevolutionary history (e.g., LZ_coev_ vs. WT_coev_, LZ_random_ vs. WT_coev_, LZ_coev_ vs. WT_random_, and LZ_random_ vs. WT_random_). There was no impact of coevolutionary history on community performance, but LacZ communities were more successful than wild‐type communities. Dotted line (*y *=* *1) indicates where relative performance, between strains, is equal. Tops and bottoms of the bars represent the 75th and 25th percentiles of the data, the white lines are the medians, and the whiskers extend from their respective hinge to the smallest or largest value no further than 1.5 * interquartile range

To see whether a shared coevolutionary history altered community performance, we competed communities where both morphs were present in both the wild‐type and LacZ in all combinations of coevolutionary history (Table 1, Figure 1c). Coevolutionary history of LacZ or wild‐type communities did not influence relative community performance in the competition trials (Figure 3). Despite the fact that the interaction between LacZ and wild‐type coevolutionary history was significant (ANOVA comparing models with and without the interaction: $\chi_{1,6}^{2}$ = 5.25, *p* = 0.02, Table S2), all Tukey's post hoc pairwise comparisons between combinations of LacZ and wild‐type coevolutionary history were non‐significant (Table 2). However, as in the relative fitness of genotypes, LacZ communities had higher relative performance than wild‐type communities (LacZ community relative performance: 1.18, 95% CI: 1.00–1.36), although it is not possible to determine whether the synergism between LacZ SM and WS contributed to this.

**Table 1:** Combinations of competition trials altering the presence and absence of coevolved or randomly assembled genotypes

Number of wild‐type morphs	Number of LacZ morphs	Wild‐type coevolutionary history	LacZ coevolutionary history
1	1	–	–
2	1	Coevolved	–
		Random	–
1	2	–	Coevolved
		–	Random
2	2	Coevolved	Random
		Coevolved	Coevolved
		Random	Coevolved
		Random	Random

*Note*

Trials were done in the presence and absence of coevolved and randomly assembled genotypes, and in situations where both morphs were present for both LacZ and wild‐type communities.

**Table 2:** Results of multiple pairwise comparisons between trials where both genotypes were present in both LacZ and wild‐type communities

Contrast			Estimate	*SE*	*df*	*t* ratio	*p*
LZ_coev_	−	LZ_random_	−0.01	0.05	47.09	−0.17	0.99
WT_coev_		WT_coev_					
LZ_coev_	−	LZ_coev_	0.05	0.05	47.09	1.00	0.75
WT_coev_		WT_random_					
LZ_coev_	−	LZ_random_	0.04	0.08	47.09	0.59	0.93
WT_coev_		WT_random_					
LZ_random_	−	LZ_coev_	0.06	0.08	47.09	0.83	0.84
WT_coev_		WT_random_					
LZ_random_	−	LZ_random_	0.05	0.05	47.09	1.00	0.75
WT_coev_		WT_random_					
LZ_coev_	−	LZ_random_	−0.01	0.05	47.09	−0.17	0.99
WT_random_		WT_random_					

*Note*.

Coevolutionary history had no impact on the outcome of community coalescence. Degrees of freedom were calculated using the Kenward–Roger method, and *p* values were adjusted using the Tukey's method for comparing a family of four estimates. Pairwise comparisons are described as trial–trial, and the composition of each input community in the trial is described. “_coev_” represents a community of genotypes that shared a coevolutionary history, and “_random_” represents a randomly assembled community of either LacZ (LZ) or wild‐type (WT).

Given that LacZ SM fitness was influenced by whether a LacZ or wild‐type WS was present, we investigated in more detail diversification of populations during the 20‐day coevolution period. Across all populations and consistent through transfers, wild‐type communities were dominated by WS morphotypes ($\bar{\text{prop}}$ = 0.97, *SD* = 0.029) whereas LacZ communities were dominated by SM ($\bar{\text{prop}}$ = 0.87, *SD* = 0.087).

## 4 DISCUSSION

Here, we experimentally investigated the effect of having a shared coevolutionary history on community coalescence. Relative fitness of a focal genotype was not affected by the presence of a coevolved genotype, but increased in the presence of a genotype of the same strain (wild‐type or LacZ). However, this effect was highly inconsistent, with only LacZ SM benefiting from the presence of LacZ WS. Unsurprisingly, given the genotype‐level results, coevolutionary history did not increase community cohesion and performance. Our results have implications for the niche‐packing hypothesis with respect to how resource partitioning occurs across allopatric populations and how evolutionary interactions within communities influence community coalescence.

Niche‐packing and resource specialization should be optimized when individuals have coevolved together, as coevolution drives adaptive radiations and character displacements (Schluter, 2000). As such, we compared the fitness of genotypes relative to the same morph (e.g., LacZ WS vs. wild‐type WS) when competed alone and in the presence and absence of morphs with which they had coevolved or another random genotype. Relative fitness was independent of coevolutionary history for all morphs, but relative fitness of LacZ SM (compared to wild‐type SM) significantly increased when in the presence of LacZ WS compared to the presence of wild‐type WS (Figure 2a). This suggests the LacZ smooth morph evolved to be locally adapted to LacZ wrinkly spreaders.

As diversification in this system is primarily driven by within‐population competition (Rainey & Travisano, 1998), the dominance of SM ($\bar{\text{prop}}$ = 0.87) suggests that SM benefited from WS by exploiting additional resources from this morph. Previous research on this experimental system shows that SM can invade WS biofilms (Zhang et al., 2009) and microbes have been shown to evolve to utilize waste products in single‐ (Helling, Vargas, & Adams, 1987) and multi‐species experimental systems (Lawrence et al., 2012). In contrast, wild‐type communities were dominated by the WS morphotype ($\bar{\text{prop}}$ = 0.97), suggesting a lack of evolved interactions between morphs as WS fitness is independent of its community (Hodgson et al., 2002). Differences in evolutionary trajectories between LacZ and wild‐type communities were likely driven by the LacZ marker itself, which was not found to be neutral as previously reported (Zhang & Rainey, 2007). However, the experimental design means that any effect of coevolutionary history on community coalescence would have been observed regardless of the consistent fitness advantage of the LacZ genotypes and communities (i.e., we would expect a greater advantage when the LacZ genotypes had coevolved together compared to when they were randomly assembled). Consequently, we do not think it had a significant impact on the conclusions of the study. The asymmetric impact of morphotype presence on relative fitness highlights the importance of local adaptation when studying community interactions.

Community performance was independent of whether constituent or competing morphotypes shared a coevolved history, suggesting that the efficiency of resource use between communities closely resembled one another (Tikhonov, 2016). Consequently, coevolved communities were not more cohesive than randomly assembled communities when coalesced and this resulted in a chimeric community outcome (coexistence). This suggests that randomly assembled communities had similar within‐community competition to that of coevolved communities, making morphotypes interchangeable between communities with the same strain background (wild‐type or LacZ). As in previous studies using *P. fluorescens*, the niche partitioning between WS and SM morphotypes (co)evolved in parallel across allopatric communities (Bailey, Rodrigue, & Kassen, 2015; McDonald, Gehrig, Meintjes, Zhang, & Rainey, 2009; Rainey & Travisano, 1998). However, our results suggest that the niche partitioning is at least partially distinct between LacZ and wild‐type strains.

Abiotic factors and the conditions of coalescence also likely contributed to the consistent coexistence between mixed communities. Our trials were done under equal ratios (50:50) of communities into fresh media, an abiotic environment that both strains evolved in for 20 days. Thus, priority effects which can result in the resident community dominating are minimized (Devevey, Dang, Graves, Murray, & Brisson, 2015; Kennedy, Peay, & Bruns, 2009; Knope, Forde, & Fukami, 2012; Peay, Belisle, & Fukami, 2011). In the absence of these effects, we demonstrated that communities can coexist through equal opportunity for niche space. However, theoretical (Vanoverbeke, Urban, & De Meester, 2016) and empirical studies (Rummens et al., 2018; Tucker & Fukami, 2014) would suggest that the presence of such effects (unequal ratios, priority effects and local adaptation) could result in community asymmetry in coalescence. Similarly, how a community shapes its environment, prior to coalescence, could benefit or destabilize the resident or invader community, depending on the constituent species’ ecological requirements (Grman & Suding, 2010; Rummens et al., 2018). How different coalescence conditions (i.e., timing, mixing ratios and environmental conditions) influence community resource use and thereby the outcome of community coalescence is a promising avenue of future research (Rillig et al., 2015).

For this study, we used a highly simplified experimental system to study the impact of (co)evolutionary history in the outcome of community coalescence. This allowed us to control the complexity in the experiment, but most importantly guaranteed us genotypes that came from a coexisting population that had shared a (co)evolutionary history. This approach has been used previously to study evolution and the maintenance of ecological diversity (Gómez & Buckling, 2013; Rainey & Travisano, 1998), but this is the first study using it to study between‐community interactions. Complementary approaches to study community coalescence use larger, more complex communities and add or remove abiotic and biotic variables (Livingston et al., 2013; Lu et al., 2018; Rummens et al., 2018; Sierocinski et al., 2017). As microbial community coalescence remains poorly understood, both experimental approaches will be invaluable for understanding and predicting coalescence outcomes.

In conclusion, our results suggest that coevolutionary history may not influence the outcome of community coalescence, at least when (co)evolution occurs in parallel. The importance of these specific (co)evolutionary relationships in driving performance at the community level highlights the need to consider interactions at lower organizational levels when studying community coalescence.

## AUTHOR CONTRIBUTION

D.P., A.B. and M.C. conceived the study and designed the experimental work. D.P. and M.C. conducted the experiments and D.P. analysed the data. All authors contributed equally to the writing of the manuscript.

## Data Availability

All data and R code used in the is available on GitHub: https://bit.ly/2NKTkCA and is archived on Zenodo: https://doi.org/10.5281/zenodo.1464583.
