# Supplementary material for: A shared coevolutionary history does not alter the outcome of coalescence in experimental populations of Pseudomonas fluorescens
Source: J Evol Biol. 2018 Nov 12;32(1):58–65. doi: 10.1111/jeb.13394 (PMC7379710; doi:10.1111/jeb.13394)
Supplement: Supplementary file 1 [file JEB-32-58-s001.pdf]

Appendix 1 – Supplementary material

Table S1. Competition trials for a single block of community pairings of LacZ and wild-type genotypes (WS: wrinkly-spreader, SM: smooth) and the total number of replicates of each treatment. Numbers identify morphs of shared coevolutionary histories.

Treatment	LacZ		wild-type		Total replicates
1 genotype *vs.* 1 genotype	WS1	-	WS2	-	12
	WS3	-	WS4	-	
	-	SM1	-	SM2	
	-	SM3	-	SM4	
2 coevolved genotypes *vs.* 1 genotype	WS1	SM1	WS2	-	24
	WS3	SM3	WS4	-	
	WS1	-	WS2	SM2	
	WS3	-	WS4	SM4	
	WS1	SM1	-	SM2	
	WS3	SM3	-	SM4	
	-	SM1	WS2	SM2	
	-	SM3	WS4	SM4	
2 random genotypes *vs.* 1 genotype	WS1	SM3	WS2	-	48
	WS3	SM1	WS2	-	
	WS1	SM3	WS4	-	
	WS3	SM1	WS4	-	
	WS1	SM3	-	SM2	
	WS3	SM1	-	SM2	
	WS1	SM3	-	SM4	
	WS3	SM1	-	SM4	

	WS1	-	WS2	SM4	
	WS1	-	WS4	SM2	
	WS3	-	WS2	SM4	
	WS3	-	WS4	SM2	
	-	SM1	WS4	SM2	
	-	SM1	WS2	SM4	
	-	SM3	WS4	SM2	
	-	SM3	WS2	SM4	
2 coevolved genotypes *vs.* 2 random genotypes	WS1	SM3	WS2	SM2	24
	WS3	SM1	WS2	SM2	
	WS1	SM3	WS4	SM4	
	WS3	SM1	WS4	SM4	
	WS1	SM1	WS2	SM4	
	WS1	SM1	WS4	SM2	
	WS3	SM3	WS2	SM4	
	WS3	SM3	WS4	SM2	
2 coevolved genotypes *vs.* 2 coevolved genotypes	WS1	SM1	WS2	SM2	12
	WS3	SM3	WS4	SM4	
	WS1	SM1	WS4	SM4	
	WS3	SM3	WS2	SM2	
2 random genotypes *vs.* 2 random genotypes	WS1	SM3	WS2	SM4	12
	WS3	SM1	WS2	SM4	
	WS1	SM3	WS4	SM2	
	WS3	SM1	WS4	SM2	

Table S2. Results of the linear mixed effects model analysis for the effect of coevolutionary history on relative community performance in communities with equal functional diversity. Results of model selection highlighted in bold.

Model	d.f.	AIC	LogLik	χ^2	P
Random effects structure					
random = ~1 | block					
Fixed effects structure					
**1. relative community performance ~ 1 + coev history of LacZ * coev history of wild-type**	6	-16.89	14.45		
2. relative community performance ~ 1 + coev history of LacZ + coev history of wild-type	5	-13.64	11.82	5.25	**0.02**

Table S3. Results of multiple pairwise comparisons of relative fitness of genotypes in the presence and absence of an additional genotype. Inconsistent differences were observed, with LacZ SM doing better when in the presence of any LacZ WS. Degrees of freedom were calculated using the Kenward-Roger method and p values were adjusted using the Tukey method for comparing a family of 6 estimates. Significant contrasts are highlighted in bold. Pairwise comparisons are presented as trial combination – trial combination and the composition of each trial is described in terms of the LacZ (LZ) and wild-type (WT) morphotypes that are present in each trial.

contrast	estimate	SE	d.f.	t ratio	p
smooth morphotype:					
**LZ SM WT SM & WS - LZ SM & WS WT SM**	**-0.45**	**0.11**	**62.48**	**-4.18**	**0.0003**
LZ SM WT SM - LZ SM WT SM & WS	0.19	0.13	62.48	1.45	0.32
LZ SM WT SM - LZ SM & WS WT SM	-0.26	0.13	62.48	-1.96	0.13
wrinkly spreader morphotype:					
LZ WS WT WS & SM - LZ WS & SM WT WS	-0.08	0.11	62.48	-0.76	0.73
LZ WS *vs.* WT WS - LZ WS WT WS & SM	0.006	0.13	62.48	0.05	0.99
LZ WS WT WS - LZ WS & SM WT WS	-0.08	0.13	62.48	-0.57	0.83
